# On Plastic Fillings

**Published:** 1889-05

**Authors:** D. D. Atkinson

**Affiliations:** Brunswick, Ga.


ARTICLE VI.

### ON PLASTIC FILLINGS.

BY D. D. ATKINSON, D. D. S., BRUNSWICK, GA.

There is, perhaps, no class of filling materials which has a more useful field than the plastic fillings, now used and endorsed by the profession. Although he did not succeed in transferring American dentistry from the "gold basis" to that of the "New Departure;" there is, perhaps, no man who has done more for the restoration to usefulness of weak and broken teeth, than has Dr. J. Foster Flagg, in his efforts to bring before the profession the great virtue in such materials. We find merit and demerit in all which have been as yet put upon the market, because this wonderful process has not attained to its possibilities. The zinc phosphates, either separate or in combination with other material, have a most useful application. With them shells of incisors and bicuspids may be filled and preserved for years, while to fill them with gold would be impossible. Yet, therefore are conditions under which very frail teeth may be first filled with cement to give body, and finished with gold, and become almost as strong as one with slight decay.

About eight years ago, I had occasion to build up two central incisors in the same mouth. One of which was an ordinary contour filling, with strong walls. The other had lost so much of the tooth substance that the red gum was plainly visible through the thin walls. This tooth, I first filled with cement, then shaped the cavity in it just as I would were it all dentine, and leaving no part of the thin enamel unprotected by it except on the face edge. The gold was packed ordinarily; and that tooth is to-day in good condition, equal to its fellow-central in beauty and usefulness. Indeed, such combinations are made every day in my practice; and never do I fill a deep cavity, where the pulp is intact with either gold or amalgam, without first placing over the bottom a layer of some non-conducting filling material. Gutta-percha is often very suitable.

There are teeth wherein caries has progressed so far that to remove all of the soft decomposed tissue would expose the pulp and slight pressure would cause pain. That last layer of decomposed dentine is the very best cap that pulp could have, and consequently must not be moved. To floor such a cavity with gutta-percha would be impracticable, for the pressure necessary to place it, however slight, would cause an encroachment of the thin layer of decomposed dentine upon the pulp. This, of course, would end very soon in pericementitis or abscess. In such cases cement is indicated. The cavity must be dried, treated with some germacide, then dried again ; a few gentle blasts from the hot air syringe are very effective, and the cement, mixed quite thin, placed and allowed to settle in position, avoiding any pressure. After this has set, the tooth may be filled with any approved material and promise good results. I believe all of the cements I have ever seen will dissolve at the cervical wall, if the cavity extends under the gum. This is very unfortunate, for that is the place above all others that the tooth needs protection. In that case, if it is desirable that the tooth be filled with cement, I would, if possible, fill over the cervical wall and some distance up with gutta-percha, or, perhaps, with amalgam, and finish with cement. These combinations carry with them symmetry and durability; gutta-percha cannot support the wall, but must be supported by the walls. Cement does, in a large measure, support sometimes a frail wall, but it will dissolve under the gum. Gutta-percha will not, hence it is a good combination. First, put the gutta-percha at the cervical wall and then fill with cement to support the frail enamel of the walls and to preserve the natural appearance of the teeth.

It may be necessary to overlook these fillings at intervals, but the same can be said of our most permanent fillings. How often in the examination of teeth which have been previously filled with cement, do we find the coronal portion in a perfect state of preservation? While at the cervical portion, the cement has all dissolved and left the pulp exposed, while the patient is not even aware that any part of the filling is gone. This may be expected when cement alone is used, and in almost every instance, when the cavity extends under the gum; but ordinarily, if the cavity is well prepared and a good article of gutta-percha carefully placed at the cervical wall, and the filling finished with cement, it may be relied upon to remain intact after the coronal portion of the filling has worn away. The reader will observe that I speak of those teeth in which gold cannot be placed on account of frail enamel only being left, and in which amalgam cannot be placed on account of the color it would produce.—*Southern Dental Journal.*

